# A System of Obstetric Medicine and Surgery, Theoretical and Clinical

**Published:** 1886-01

**Authors:** 


A SYSTEM OF OBSTETRIC MEDICINE AND SURGERY, THEORETICAL AND CHEMICAL. For the student and practitioner. By Robert Barnes, M. D., Obstetric Physician to St. George's Hospital; Consulting Physician to the Chelsea Hospital for Women, etc., etc.; and Fancourt Barnes, M. D., Physician to the

Royal Maternity and to the British Lying-in Hospital; Assistant Obstetric Physician to the Great Northern Hospital; Physician to the Chelsea Hospital for Women. Illustrated with two hundred and thirty-one wood-cuts. Philadelphia: Lea Brothers & Co. 1885.

The deservedly high standing of the senior editor of this book, with the fair reputation of the junior editor, and the prominence of the third co-laborer in the embyological department of the work, must secure for this systematic treatise due consideration from the American obstetrician and gynecologist. The reader cannot fail to discern the dogmatic style which springs from a sense of self-reliance, but which, in this instance, as in others marked by great minds, lessens the value of the production in the estimate of those who weigh properly the facts and meaning of the author.

When the authors attribute the so-called hour-glass contraction to constriction of the lower segment of the womb, the notorious fact of the comparative relaxation of all below the contracted band seems to have escaped their attention.

In treating of the difficulties of turning in the advanced stage of face presentations, it evidently has not occurred to them that their embarrassment ceases with the resort to extreme anæsthesia for inducing uterine relaxation. Like want of recognition of well-established principles in obstetric and gynecological practice will be detected by the discriminating reader; and yet, withal, the work comes more nearly up to the requirements of scientific midwifery than any previous production.

